# A mathematical model of visceral leishmaniasis transmission and control: Impact of ITNs on VL prevention and elimination in the Indian subcontinent

**DOI:** 10.1371/journal.pone.0311314

**Published:** 2024-10-04

**Authors:** Cameron Davis, Elizabeth R. Javor, Sonja I. Rebarber, Jan Rychtář, Dewey Taylor

**Affiliations:** 1 Department of Mathematics, Fitchburg State University, Fitchburg, MA, United States of America; 2 Department of Mathematics, Rochester Institute of Technology, Rochester, NY, United States of America; 3 Department of Mathematics and Statistics, Swarthmore College, Swarthmore, PA, United States of America; 4 Department of Mathematics and Applied Mathematics, Virginia Commonwealth University, Richmond, VA, United States of America; Mashhad University of Medical Sciences, IRAN, ISLAMIC REPUBLIC OF

## Abstract

Visceral Leishmaniasis (VL) is a deadly, vector-borne, parasitic, neglected tropical disease, particularly prevalent on the Indian subcontinent. Sleeping under the long-lasting insecticide-treated nets (ITNs) was considered an effective VL prevention and control measures, until KalaNet, a large trial in Nepal and India, did not show enough supporting evidence. In this paper, we adapt a biologically accurate, yet relatively simple compartmental ordinary differential equations (ODE) model of VL transmission and explicitly model the use of ITNs and their role in VL prevention and elimination. We also include a game-theoretic analysis in order to determine an optimal use of ITNs from the individuals’ perspective. In agreement with the previous more detailed and complex model, we show that the ITNs coverage amongst the susceptible population has to be unrealistically high (over 96%) in order for VL to be eliminated. However, we also show that if the whole population, including symptomatic and asymptomatic VL cases adopt about 90% ITN usage, then VL can be eliminated. Our model also suggests that ITN usage should be accompanied with other interventions such as vector control.

## Introduction

Visceral Leishmaniasis (VL) is a deadly, vector-borne, parasitic, neglected tropical disease found primarily in the Indian Subcontinent, East Africa, and Brazil [1]. In the Indian subcontinent, which accounts for over two thirds of VL cases [2], VL is caused by parasites belonging to Leishmania donovani complex [3]. There is no known animal reservoir and parasites are transmitted from human to human by female sandfly *Phlebotomous argentipes* [4].

Because VL treatment is expensive and sometimes ineffective [5], VL prevention is critical. There is currently no available vaccine [6, 7] and the prevention focuses on active case detection [8], vector control [9], and bite prevention [10]. The efficacy of long-lasting insecticide-treated nets (ITNs) in the prevention of VL was evaluated by KalaNet, a cluster randomized controlled trial in Nepal and India [11]. Since the distribution of ITNs during the KalaNet trial did not reduce the risk of L. donovani infection or clinical VL [12], ITNs are not part of the standard government VL control programme in India [13].

Mathematical modeling is now a common and necessary tool used to understand epidemics and disease elimination efforts [14, 15]. In contrast to most neglected tropical diseases [16], there are many different models of VL; see for example [17–20] for recent reviews. A comprehensive model of VL for the Indian Subcontinent was developed in [21]. [13] built on [21] to quantify the role of ITN use in VL transmission. They have shown that in order to eliminate VL, the ITN usage would have to be above 96%. For comparison, at the end of KalaNet trial, the ITNs use was still only around 91% [12].

The model of [13] included twelve human and three sandfly compartments and a long list of parameters. The purpose of this paper is to use a simpler model with a shorter list of parameters to see whether this could yield to different predictions. This is in line with [22] who discusses the tension between model realism and mathematical tractability and proposed rules for simplifying the models in an approach that can be particularly beneficial for modeling diseases affecting developing countries. We also explicitly investigate how the ITNs usage amongst the infected population influences the outcomes.

## Model

We adopt a model with seven human and two sandfly compartments from [17] and extend in by a game-theoretic component which incorporates the voluntary use of insecticide treated nets (ITNs) as in [13]. This significantly simplifies the model from [13] while still being in line with a biologically realistic model from [17]. The underlying disease transmission model makes all of the standard assumptions and has all of the typical limitations of compartmental models as discussed, e.g., in [23].

### Disease transmission model

We will consider the human and sandfly population. The human population is divided into seven groups: susceptible (*S*), exposed (*E*), infected asymptomatic (*I*_*A*_), infected symptomatic (also called Kala-Azar, *I*_*K*_), infected dormant (*I*_*D*_), PKDL (*I*_*P*_) and fully recovered individuals (*R*). The total human population is *N* where *N* = *S* + *E* + *I*_*A*_ + *I*_*K*_ + *I*_*D*_ + *I*_*P*_ + *R*.

The sandfly population is divided into susceptible (*S*_*F*_) and infectious (*I*_*F*_). The total sandfly population is *N*_*F*_ = *S*_*F*_ + *I*_*F*_. For simplicity, we assume *N*_*F*_ = *n*_*F*_*N*; here *n*_*F*_ is the number of sandflies per person.

The dynamics is as follows. Human individuals are all born as susceptible at rate Λ. Susceptible individuals become exposed at a rate of

$$\begin{array}{c} \lambda =\left(1-p\right)\beta i_{F}I_{F}, \end{array}$$ (1)

where (1 − *p*) is the proportion of the human population that does not use ITNs, *β*^−1^ is the duration of the sandfly feeding cycle, and *i*_*F*_ is the probability that an infected sandfly infects a human.

After an incubation time $\gamma_{E}^{-1}$, the exposed individuals become infectious asymptomatic (*I*_*A*_). The average duration of asymptomatic infection is $\gamma_{A}^{-1}$ and the individuals can (a) develop Kala-Azar (with probability *f*_*AK*_), (b) dormant infection (with probability *f*_*AD*_), (c) PKDL (with probability *f*_*AP*_), or (d) recover (with probability *f*_*AR*_ = 1 − (*f*_*AK*_ + *f*_*AD*_ + *f*_*AP*_).

The symptomatic infection, Kala-Azar, is a serious infection that results in an additional mortality *μ*_*K*_. If we assume that the patient survived, the infection lasts for an average time $\gamma_{K}^{-1}$. With probability *f*_*KD*_, the symptoms resolve and the individual moves to a dormant infection stage; with probability 1 − *f*_*KD*_, the individuals fully recover.

Cases with dormant infection develop PKDL after an average time $\gamma_{D}^{-1}$ (assuming they survived). After the PKDL infection which lasts $\gamma_{P}^{-1}$ on average, the individuals recover. Recovered individuals maintain immunity for time *ρ*^−1^, after which they become susceptible.

Independent of VL, humans die at a rate *μ* from every compartment. As mentioned above, cases with Kala-Azar suffer from an additional mortality rate *μ*_*K*_.

For simplicity, we assume that the sandfly population remains constant *N*_*F*_. The flies live for an average time $\mu_{F}^{-1}$ and are born at rate *μ*_*F*_*N*_*F*_ as susceptible.

The susceptible sandflies become infected at rate

$$\begin{array}{c} \lambda_{F}=\beta (i_{A}\left(1-p_{A}\right)I_{A}+i_{K}\left(1-p_{K}\right)I_{K}+i_{D}\left(1-p_{D}\right)I_{D}+i_{P}\left(1-p_{P}\right)I_{P}), \end{array}$$ (2)

where *β*^−1^ is the duration of the feeding cycle, and, for *x* ∈ {*A*, *K*, *D*, *P*}, *i*_*x*_ is the proportion of sandfly bites of humans in infected compartment *I*_*x*_ that transmits the VL infection, and *p*_*x*_ is the proportion of cases in compartment *I*_*x*_ that uses ITNs.

**Fig 1:**
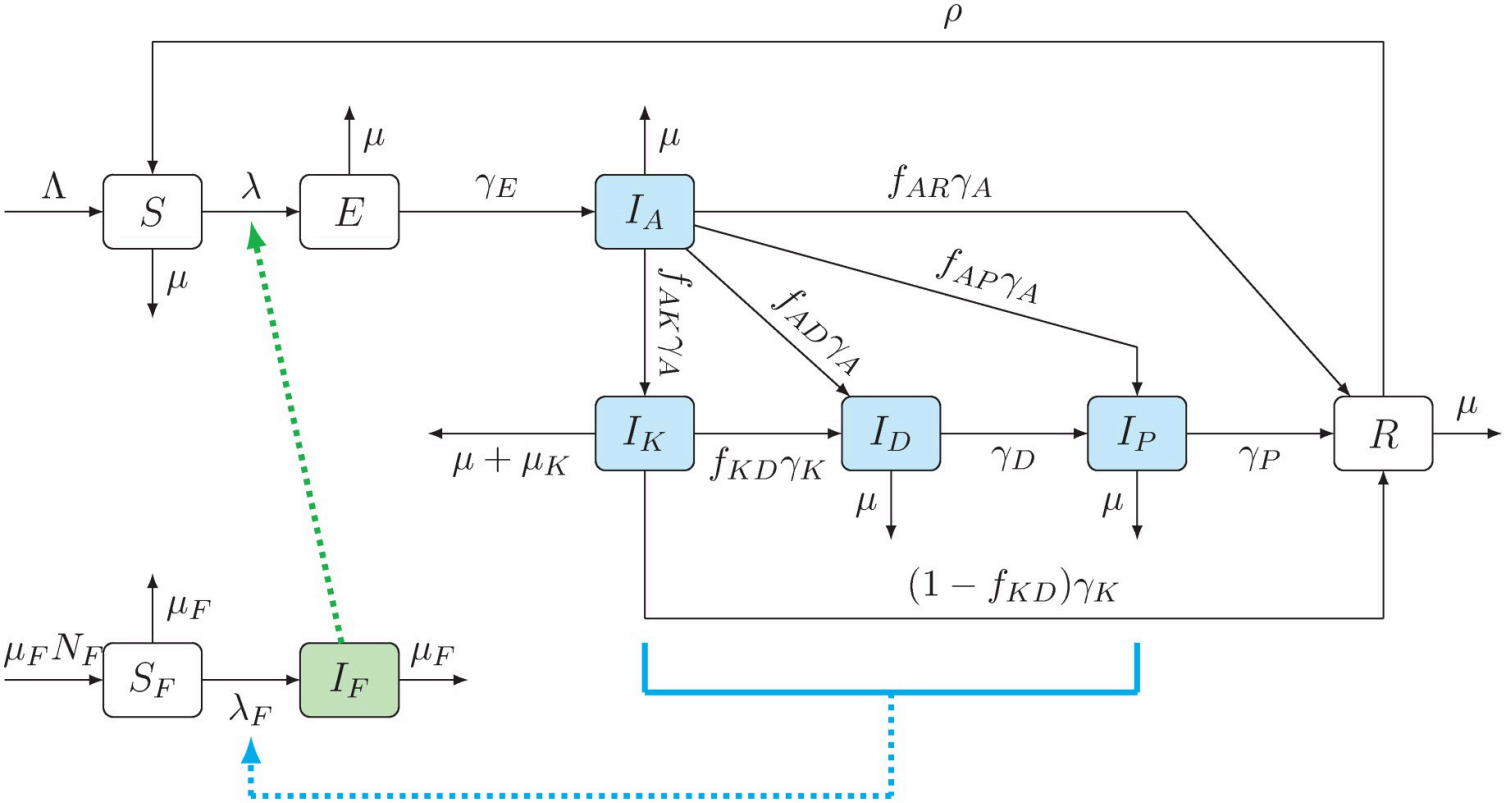
Compartmental model of VL transmission dynamics; based on [17]. Human population is shown in the top seven compartments, and sandflies at the bottom two. Solid arrows represent transitions into and out of each compartment, the formulas next to the arrows are the transmission rates. All humans are born as susceptible (*S*). They become exposed (*E*) at rate *λ* = (1 − *p*)*βi*_*F*_*I*_*F*_ given in (1) when bitten by an infectious sandfly (*I*_*F*_). The green dotted line represents this causation. Exposed cases then become asymptomatic (*I*_*A*_). The asymptomatic cases can develop Kala-Azar, become dormant (*I*_*D*_), develop PKDL (*I*_*P*_) or recover (*R*). The Kala-Azar cases can either recover directly, or progress through dormant and PKDL stages before finally recovering. The recovered cases eventually lose immunity and become susceptible again. Sandflies are born susceptible (*S*_*F*_), and can become infectious (*I*_*F*_) at rate *λ*_*F*_ given by (2) by biting an infected human (in *I*_*A*_, *I*_*K*_, *I*_*D*_, *I*_*P*_). The blue dotted line represents this causation.

The dynamic is summarized in Fig 1. The notation is summarized in Tables 1 and 2.

**Table 1:** Parameter values. Times are in months and the rates are *per capita* per month. Detailed derivation can be found in [13]; here we also list original sources for the reference. “Range” is the range of values used in sensitivity analyses. The value of *f*_*AR*_ is determined by 1 − (*f*_*AD*_ + *f*_*AK*_ + *f*_*AP*_).

Symbol	Meaning	Value	Range	Reference(s)
*n*_*F*_	Number of sandflies per human	3	[2, 3.5]	[24]
*i*_*F*_	Probability that a bite by an infected sandfly infects a susceptible human	1	[0, 1]	[25]
$\mu_{F}^{-1}$	Expected lifespan of sandflies	$\frac{14}{30}$	$\left[\frac{6}{30},\frac{31}{30}\right]$	[26]
*β* ^−1^	Feeding cycle duration	$\frac{4}{30}$	$\left[\frac{3}{30},\frac{8}{30}\right]$	[27]
Λ	Birth rate	$\frac{0.0277}{12}$	$\left[\frac{0.025}{12},\frac{0.030}{12}\right]$	[28]
*μ* ^−1^	Life expectancy	67.8 * 12	[65, 72] * 12	[29]
$\mu_{K}^{-1}$	Life expectancy with KA	30	[5, 36]	[30]
*p*	Percentage of *S* using ITNs	0.7	[0.5, 1]	[31]
*p*_*A*_	Percentage of *I*_*A*_ using ITNs	0.7	[0.6, 0.8]	[31]
*p*_*K*_	Percentage of *I*_*K*_ using ITNs	0.7	[0.6, 0.8]	[31]
*p*_*D*_	Percentage of *I*_*D*_ using ITNs	0.7	[0.6, 0.8]	[31]
*p*_*P*_	Percentage of *I*_*P*_ using ITNs	0.7	[0.6, 0.8]	[31]
$\gamma_{E}^{-1}$	Incubation period	4	[1, 10]	[32]
$\gamma_{A}^{-1}$	Sojourn time in *I*_*A*_	6	[4.5, 10]	[33]
$\gamma_{K}^{-1}$	Sojourn time in *I*_*K*_	2	[1, 3.5]	[34]
$\gamma_{D}^{-1}$	Sojourn time in *I*_*D*_	21	[16, 26]	[35]
$\gamma_{P}^{-1}$	Sojourn time in *I*_*P*_	30	[17, 90]	[36]
*f*_*KD*_	Fraction of *I*_*K*_ who become dormant	0.063	[0.02, 0.1]	[21]
*f*_*AK*_	Fraction of *I*_*A*_ who develop KA	0.035	[0.01, 0.15]	[37]
*f*_*AD*_	Fraction of *I*_*A*_ who become dormant	5.5 * 10^−4^	[10^−4^, 10^−3^]	[35]
*f*_*AP*_	Fraction of *I*_*A*_ who develop PKDL	6 * 10^−5^	[10^−5^, 10^−4^]	[38]
*f*_*AR*_	Fraction of *I*_*A*_ who become recovered	0.96439		Fixed
*ρ* ^−1^	Duration of immunity	39	[14, 46]	[39]
*i*_*A*_	Probability that *I*_*A*_ infects a sandfly	0.06	[0, 0.1]	[13]
*i*_*K*_	Probability that *I*_*K*_ infects a sandfly	0.05	[0, 0.1]	[40]
*i*_*D*_	Probability that *I*_*D*_ infects a sandfly	0	[0, 0.1]	[13]
*i*_*P*_	Probability that *I*_*P*_ infects a sandfly	0.0902	[0, 0.1]	[41]
*C*_*VL*_	Cost of VL	100	[50, 150]	[13]
*C*_*ITN*_	Cost of acquiring ITN	5	[3, 7]	[42]

**Table 2:** Additional notation used.

Notation	Description	Equation
λ	Force of infection (vectors infecting humans)	(1)
λ_*F*_	Force of infection (humans infecting vectors)	(2)
*T*_*Comp*_	Expected time an individual spends in a compartment *Comp* ∈ {*E*, …, *R*} given it started in compartment *E*	(7)–(15)
*T*_*Cycle*_	Expected time it takes an individual to become susceptible again (given it started in *E* and conditional on surviving)	(14)
*T*_*I*_	Average time an individual spends as infectious to sandfly (weighted by the infectivity) *E*	(6)

### Game-theoretic component of the model

We incorporate the game-theoretic component into the above transmission dynamics in the same way as done, for example, in [43].

A game is played by susceptible individuals who decide whether or not to use ITNs. As typical in these models [44–48], we assume that players are rational, act in their own self-interest, have complete information about VL, and consider only financial costs of ITNs. The players evaluate prospective costs and benefits of their options (to use or to not use the ITNs) in relation to the actions taken by others and they choose the option that maximizes their own net payoffs (benefits minus costs).

If an individual uses an ITN, they are protected against VL, but they also incur a cost of acquiring the ITN, *C*_*ITN*_. On the other hand, when they do not use the ITN, they risk contracting VL, i.e., and, eventually, paying the cost *C*_*VL*_.

More specifically, let us now focus on a single individual deciding whether to use the ITN when the rest of the population uses ITNs with probability $\bar{p}$. If the population is large enough, the decision of a single individual will not have a significant impact on the disease transmission. We may thus assume that the number of infected flies $I_{F}^{*}$ in the population does not depend on the choice of the focal individual. As in [49–51], the risk for an unprotected susceptible individual becoming exposed (rather than staying susceptible and die of natural causes) is given by $\frac{\beta i_{F}I_{F}^{*}}{\beta i_{F}I_{F}^{*}+\mu }$. Similarly, the probability to become exposed if the individual uses ITN for the fraction *p* of the time is $\frac{\left(1-p\right)\beta i_{F}I_{F}^{*}}{\left(1-p\right)\beta i_{F}I_{F}^{*}+\mu }$. In aggregate, if an individual’s ITN usage is *p*, the cost is given by

$$\begin{array}{c} C\left(p\right)=\{\begin{array}{cc} \frac{\left(1-p\right)\beta i_{F}I_{F}^{*}}{\left(1-p\right)\beta i_{F}I_{F}^{*}+\mu }C_{VL}+C_{ITN}, & \text{if}\;0<p\leq 1,\\ \frac{\beta i_{F}I_{F}^{*}}{\beta i_{F}I_{F}^{*}+\mu }C_{VL}, & \text{if}\;p=0. \end{array} \end{array}$$ (3)

We note that above, the number of infected sandflies in the endemic equilibrium, $I_{F}^{*}$ depends on $\bar{p}$, the average ITN use in the population. Consequently, *C*(*p*) depends on $\bar{p}$ as well.

In (3), we made several implicit assumptions. First, we assumed that the cost *C*_*VL*_ is the expected cost after getting exposed to VL. In our diagram in Fig 1, an exposed individual becomes asymptomatic with probability $\frac{\gamma_{E}}{\gamma_{E}+\mu }$ and then goes through various stages of VL, including PKDL (or recovers with probability *f*_*AR*_ without ever experiencing any symptoms and incurring any costs). To fully estimate the value of *C*_*VL*_, all of the possible stages and costs at every stage would have to be accounted for; here we simplify the model by assuming *C*_*VL*_ ≈ 100 to be in line with [13]. Second, we assumed that *C*_*ITN*_ is only the cost of ITN acquisition, and it does not involve the cost of ITN usage. A person that is using ITN, however infrequently, has to pay the acquisition cost, but once the ITN is acquired, we are assuming no more costs. Finally, we ignore the effect of ITN on the mortality of the sandflies; i.e., we assume that ITNs prevent transmissions between humans and sandflies, but otherwise do not affect the sandfly population.

## Analysis

### Equilibria of the disease dynamics

Here we show only a summary of the analysis; see S1 File for more details. There are two possible equilibria, disease-free equilibrium, *DFE*^0^ and endemic equilibrium, EE*. The disease-free equilibrium is given by

$$\begin{array}{c} DFE^{0}=(\frac{\Lambda }{\mu },0,0,0,0,0,0,n_{F}\frac{\Lambda }{\mu },0), \end{array}$$ (4)

i.e., $S_{0}=\frac{\Lambda }{\mu }=N$ and *S*_*F*_ = *n*_*F*_*N* while all other compartments are zero.

When the ITNs usage in the population is $\bar{p}$, the reproduction number, $R_{0}\left(\bar{p}\right)$, i.e., the average number of new infections caused by a single-infected individual in an otherwise susceptible population [14] is given by

$$\begin{array}{c} R_{0}\left(p\right)=\left(1-p\right)\beta^{2}i_{F}n_{F}(\frac{\Lambda }{\mu }{)}^{2}\frac{1}{\mu_{F}}T_{I}, \end{array}$$ (5)

where

$$\begin{array}{c} T_{I}=i_{A}\left(1-p_{A}\right)T_{I_{A}}+i_{K}\left(1-p_{K}\right)T_{I_{K}}+i_{D}\left(1-p_{D}\right)T_{I_{D}}+i_{P}\left(1-p_{P}\right)T_{I_{P}}, \end{array}$$ (6)

and

$$\begin{array}{c} T_{E}=\frac{1}{\gamma_{E}+\mu }, \end{array}$$ (7)

$$\begin{array}{c} T_{I_{A}}=\frac{\gamma_{E}T_{E}}{\gamma_{A}+\mu }, \end{array}$$ (8)

$$\begin{array}{c} T_{I_{K}}=\frac{f_{AK}\gamma_{A}T_{I_{A}}}{\gamma_{K}+\mu +\mu_{K}}, \end{array}$$ (9)

$$\begin{array}{c} T_{I_{D}}=\frac{f_{KD}\gamma_{K}T_{I_{K}}+f_{AD}\gamma_{A}T_{I_{A}}}{\gamma_{D}+\mu }, \end{array}$$ (10)

$$\begin{array}{c} T_{I_{P}}=\frac{\gamma_{D}T_{I_{D}}+f_{AP}\gamma_{A}T_{I_{A}}}{\gamma_{P}+\mu }. \end{array}$$ (11)

Here, *T*_*I*_ can be understood as an effective time spent as an infectious individual and *T*_*X*_, for *X* ∈ {*E*, *I*_*A*_, *I*_*K*_, *I*_*D*_, *I*_*P*_, *R*} is the average time spent in the corresponding compartment *X*.

The disease-free equilibrium is locally asymptotically stable if *R*_0_(*p*) < 1 and the endemic equilibrium is stable if *R*_0_(*p*) > 1 [52].

The endemic equilibrium is given by

$$\begin{array}{c} EE^{*}=\left(S^{*},E^{*},I_{A}^{*},I_{K}^{*},I_{D}^{*},I_{P}^{*},R^{*},S_{F}^{*},I_{F}^{*}\right), \end{array}$$ (12)

and derived and expressed in closed forms in the Section Detailed calculations. For further analysis, it is important that

$$\begin{array}{c} I_{F}^{*}=\frac{n_{F}\beta \Lambda^{2}T_{I}}{\mu^{2}T_{Cycle}\mu_{F}+\mu \beta \Lambda T_{I}}(1-\frac{1}{R_{0}}) \end{array}$$ (13)

where

$$\begin{array}{c} T_{Cycle}=T_{E}+T_{I_{A}}+\frac{\left(\mu +\mu_{K}\right)}{\mu }T_{I_{K}}+T_{I_{D}}+T_{I_{P}}+T_{R}. \end{array}$$ (14)

is the average time it takes to return to a susceptible compartment (given no death) after getting exposed to VL. In (14), *T*_*R*_ is given by

$$\begin{array}{c} T_{R}=\frac{\left(1-f_{KD}\right)\gamma_{K}T_{I_{K}}+\gamma_{P}T_{I_{P}}+f_{AR}\gamma_{A}T_{I_{A}}}{\rho +\mu }. \end{array}$$ (15)

**Fig 2:**
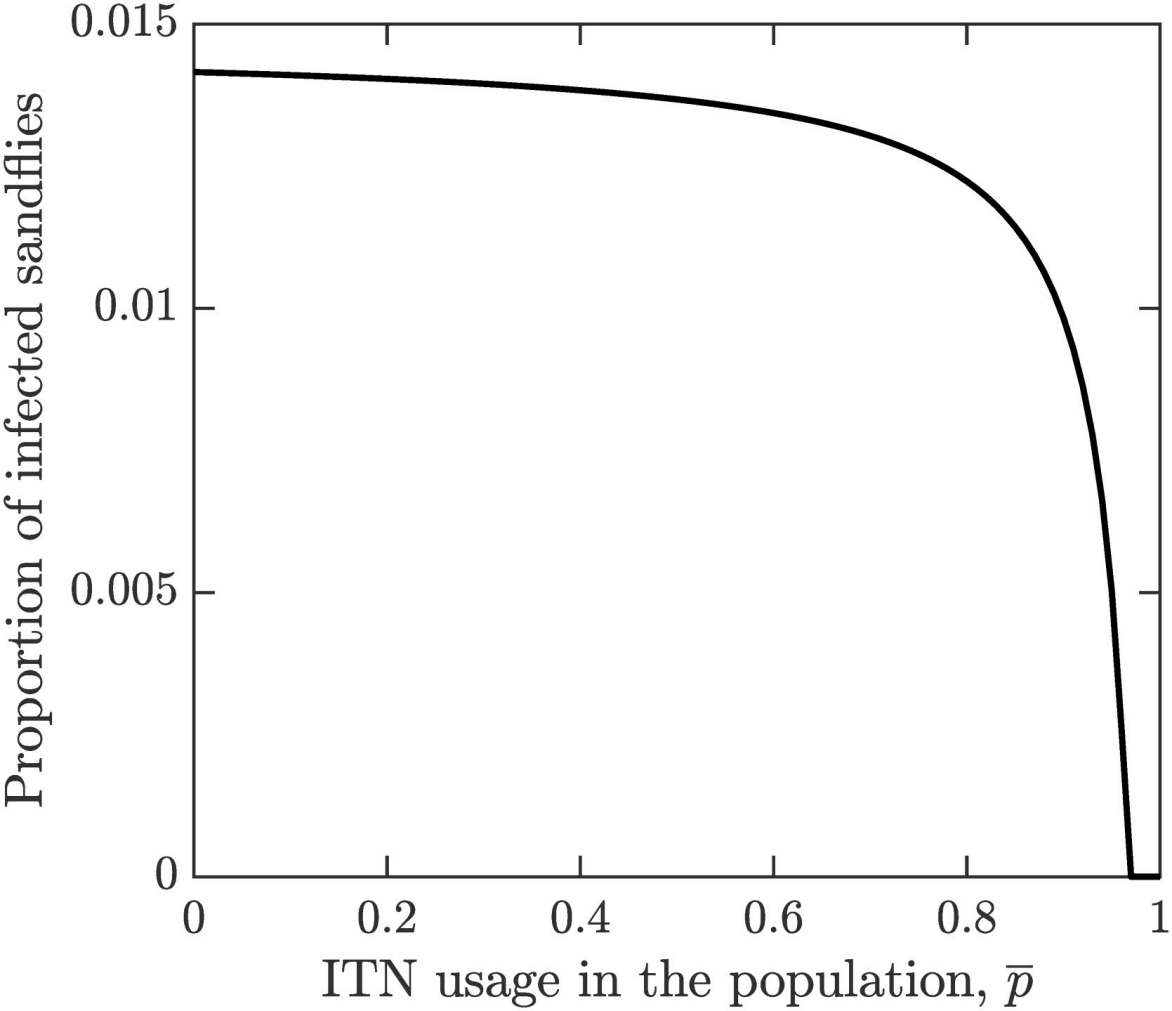
The proportion of infected sandlies. The proportion of infected sandflies in the equilibrium, $I_{F}^{*}/N_{F}$ is a decreasing function of the ITN population coverage $\bar{p}$. The parameter values are as shown in Table 1. The matlab code used to generate the figures is in S2 File.

**Fig 3:**
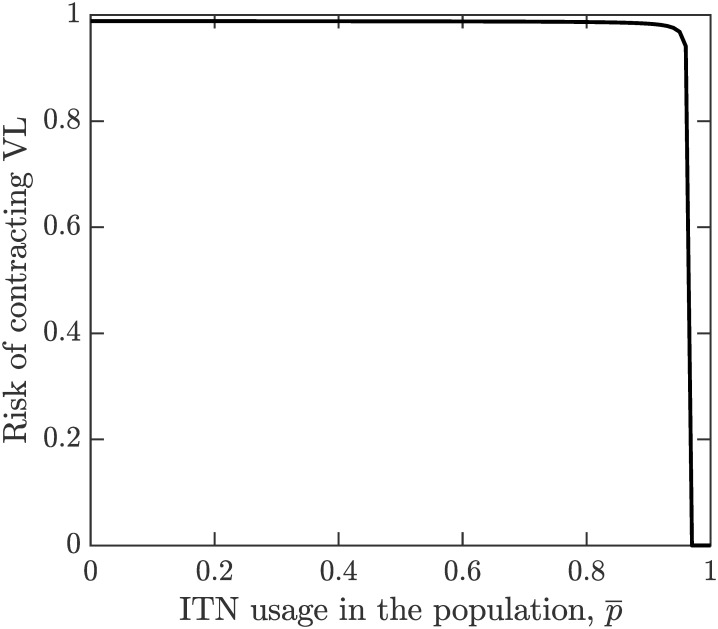
The risk of contracting VL. The risk of contracting VL, is a decreasing function of the ITN population coverage $\bar{p}$. The parameter values are as shown in Table 1.

By (5) and (13), $I_{F}^{*}$ is decreasing in $\bar{p}$. This is also shown in Fig 2. Consequently, the risk of contracting VL, $\frac{\beta i_{F}I_{F}^{*}}{\beta i_{F}I_{F}^{*}+\mu }$ also decreases with $\bar{p}$ as shown in Fig 3.

### Minimum ITN usage needed for VL elimination

The disease-free equilibrium is stable when *R*_0_(*p*) < 1. We need to find the smallest *p*_*HP*_ ∈ [0, 1] such that when *p* ≥ *p*_*HP*_ VL is in DFE. It follows from (5) that *R*_0_(*p*) = (1 − *p*)*R*_0_(0), where

$$\begin{array}{c} R_{0}\left(0\right)=\beta^{2}i_{F}n_{F}(\frac{\Lambda }{\mu }{)}^{2}\frac{1}{\mu_{F}}T_{I} \end{array}$$ (16)

is the basic reproduction number (when no one uses ITNs). As a result, the minimum ITN coverage necessary to achieve DFE is given by

$$\begin{array}{c} p_{HP}=\{\begin{array}{cc} 1-\frac{1}{R_{0}\left(0\right)} & \text{if}\;R_{0}\left(0\right)>1,\\ 0 & \text{otherwise}. \end{array} \end{array}$$ (17)

### Game-theoretic analysis

All individuals try to minimize *C*(*p*). Because

$$\begin{array}{c} C^{\prime \prime }\left(p\right)=\frac{-2{\left(\beta i_{F}I_{F}^{*}\right)}^{2}C_{VL}\mu }{{\left(\left(1-p\right)\beta i_{F}I_{F}^{*}+\mu \right)}^{3}}<0, \end{array}$$ (18)

the minimum is achieved either for *p* = 0 or *p* = 1. Therefore, the minimum value of *C*(*p*) is either $\frac{\beta i_{F}I_{F}^{*}}{\beta i_{F}I_{F}^{*}+\mu }C_{VL}$ at *p* = 0 or *C*_*ITN*_ at *p* = 1.

If $\frac{\beta i_{F}I_{F}^{*}}{\beta i_{F}I_{F}^{*}+\mu }C_{VL}>C_{ITN}$, it is in an individual’s best interest to use an ITN every night. Otherwise, they should not buy or use an ITN at all. Nash equilibrium occurs when these two costs at *p* = 0 and *p* = 1 are equal, i.e. when

$$\begin{array}{c} C_{ITN}=\frac{\beta i_{F}I_{F}^{*}}{\beta i_{F}I_{F}^{*}+\mu }C_{VL}. \end{array}$$ (19)

By (19), we must have

$$\begin{array}{c} I_{F}^{*}=\frac{\frac{C_{ITN}}{C_{VL}}\mu }{\beta i_{F}(1-\frac{C_{ITN}}{C_{VL}})}. \end{array}$$ (20)

On the other hand, by (13),

$$\begin{array}{c} I_{F}^{*}=\frac{n_{F}\beta \Lambda^{2}T_{I}}{\mu^{2}T_{Cycle}\mu_{F}+\mu \beta \Lambda T_{I}}(1-\frac{1}{R_{0}\left(0\right)\left(1-\bar{p}\right)}) \end{array}$$ (21)

and solving it for $\bar{p}$ yields

$$p_{NE}=1-\frac{1}{R_{0}\left(0\right)(1-I_{F}^{*}\frac{\mu^{2}T_{Cycle}\mu_{F}+\mu \beta \Lambda T_{I}}{n_{F}\beta \Lambda^{2}T_{I}})}$$ (22)

where $I_{F}^{*}$ is given by (20).

### Uncertainty and sensitivity analysis

For sensitivity analysis, we used the Latin hyper-cube sampling with partial rank correlation coefficient (LHS-PRCC) scheme [53, 54]. Our MATLAB code, including the code for this analysis, was uploaded as the S2 File.

## Results

For the parameter values as in Table 1, the reproduction number is *R*_0_ ≈ 9.1. At the same time, the minimum level of ITN use by the susceptible population in order for VL to be eliminated is *p*_*HP*_ ≈ 0.967 while the Nash equilibrium, i.e., the level which is optimal from the individuals’ perspective and at which no individual benefits from switching its ITN usage strategy is *p*_*NE*_ ≈ *p*_*HP*_ − 10^−5^. At the Nash equilibrium, the reproduction number is *R*_0_(*p*_*NE*_) ≈ 1.0002. This means that VL would be very close to being eliminated. These results agree with model prediction from [13]. Overall, our model also shows a good fit to the original KalaNet data as demonstrated in Table 3, again in line with [13].

**Table 3:** Comparison between KalaNet data, model from [13] and our model.

Compartment	KalaNet data	Model from [13]	Our model
*S* + *R* + *E*	0.88	0.877	0.884
*I*_*A*_	0.12	0.12	0.112
*I*_*K*_	0.00015	NA	0.00122
*I*_*D*_	NA	NA	0.001
*I*_*P*_	0.00005	NA	0.00141
*I*_*F*_	0.005	0.011	0.013
*R*_0_	NA		9.1
*p*_*HP*_	> 0.8	0.959	0.967
*p*_*HP*_ − *p*_*NE*_	NA	≈ 10^−4^	≈ 10^−5^
*R*_0_(*p*_*NE*_)	NA	1.002	1.0002

**Fig 4:**
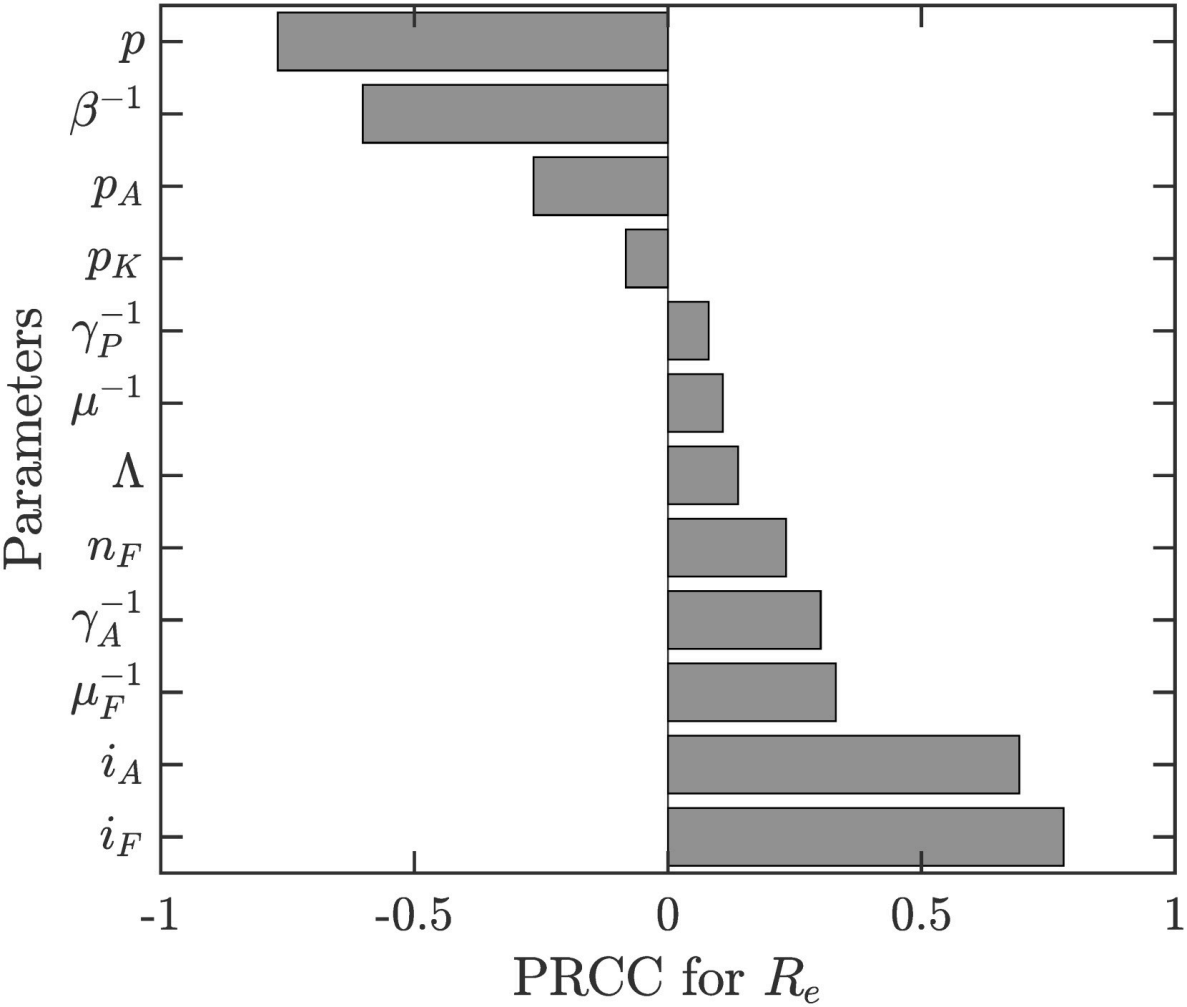
Sensitivity analysis of *R*_0_. The parameter ranges are as in Table 1. Only parameters with sensitivity index over 7.5% are shown.

**Fig 5:**
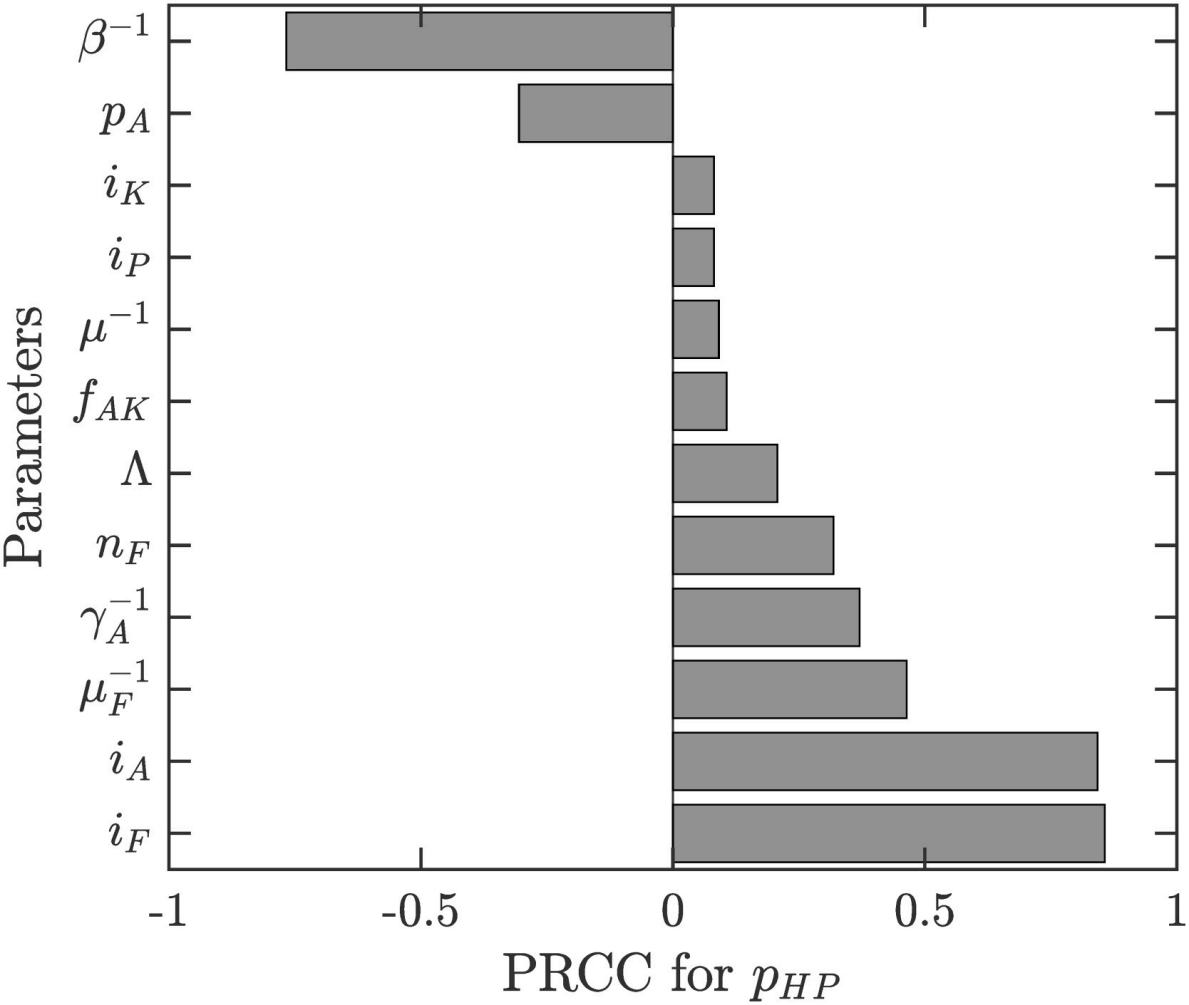
Sensitivity analysis of *p*_*HP*_. The parameter ranges are as in Table 1. Only parameters with sensitivity index over 7.5% are shown.

**Fig 6:**
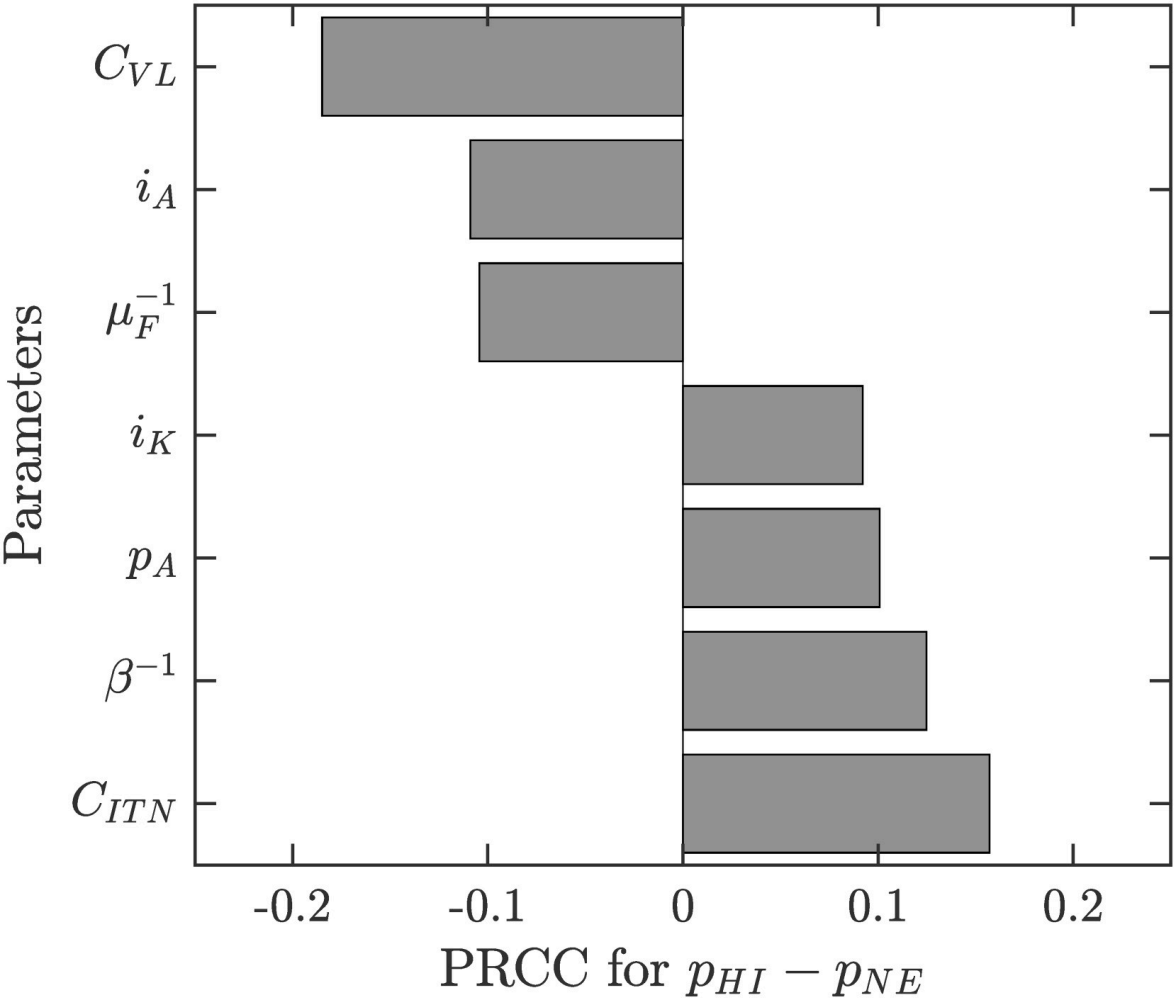
Sensitivity analysis of *p*_*HP*_ − *p*_*NE*_. The parameter ranges are as in Table 1. Only parameters with sensitivity index over 7.5% are shown.

Figs 4–6 show the sensitivity of *R*_0_, *p*_*HP*_ and *p*_*HP*_ − *p*_*NE*_ on various parameters.

We can see from Figs 4 and 5 that as the duration of the sandfly feeding cycle, *β*^−1^, increases, *R*_0_ and *p*_*HP*_ decreases. On the other hand, *R*_0_ and *p*_*HP*_ are increasing in *i*_*F*_ (the probability of VL transmission from a biting infected sandfly to humans) and *i*_*A*_ (the probability of VL transmission from asymptomatic VL cases to sandfly). Both of these parameters are also increasing in $\mu_{F}^{-1}$ (the lifespan of sandflies), $\gamma_{A}^{-1}$ (sojourn times of asymptomatic infections) and *n*_*F*_ (number of sandflies per person). The influence of other parameters is relatively negligible.

It follows from (22) that *p*_*HP*_−*p*_*NE*_ is quite small. This is also illustrated in Fig 3 by the fact that the risk of contracting VL is essentially a step-wise function with “jump” occurring at *p*_*HP*_. Fig 6 shows that the sensitivity of *p*_*HP*_ − *p*_*NE*_ on various parameter values is quite small; i.e., the difference is small regardless of parameter values. We note that the difference increases most with the cost of ITN use (*C*_*ITN*_), the duration of the feeding cycle (*β*^−1^) and the ITN usage amongst asymptomatic cases (*p*_*A*_) or the probability of VL transmission from Kala-Azar cases to sandflies (*i*_*K*_). Also, the difference is decreasing in the cost of VL (*C*_*VL*_), the probability of VL transmission of asymptomatic individuals to sandflies (*i*_*A*_) and the lifespan of sandflies ($\mu_{F}^{-1}$).

**Fig 7:**
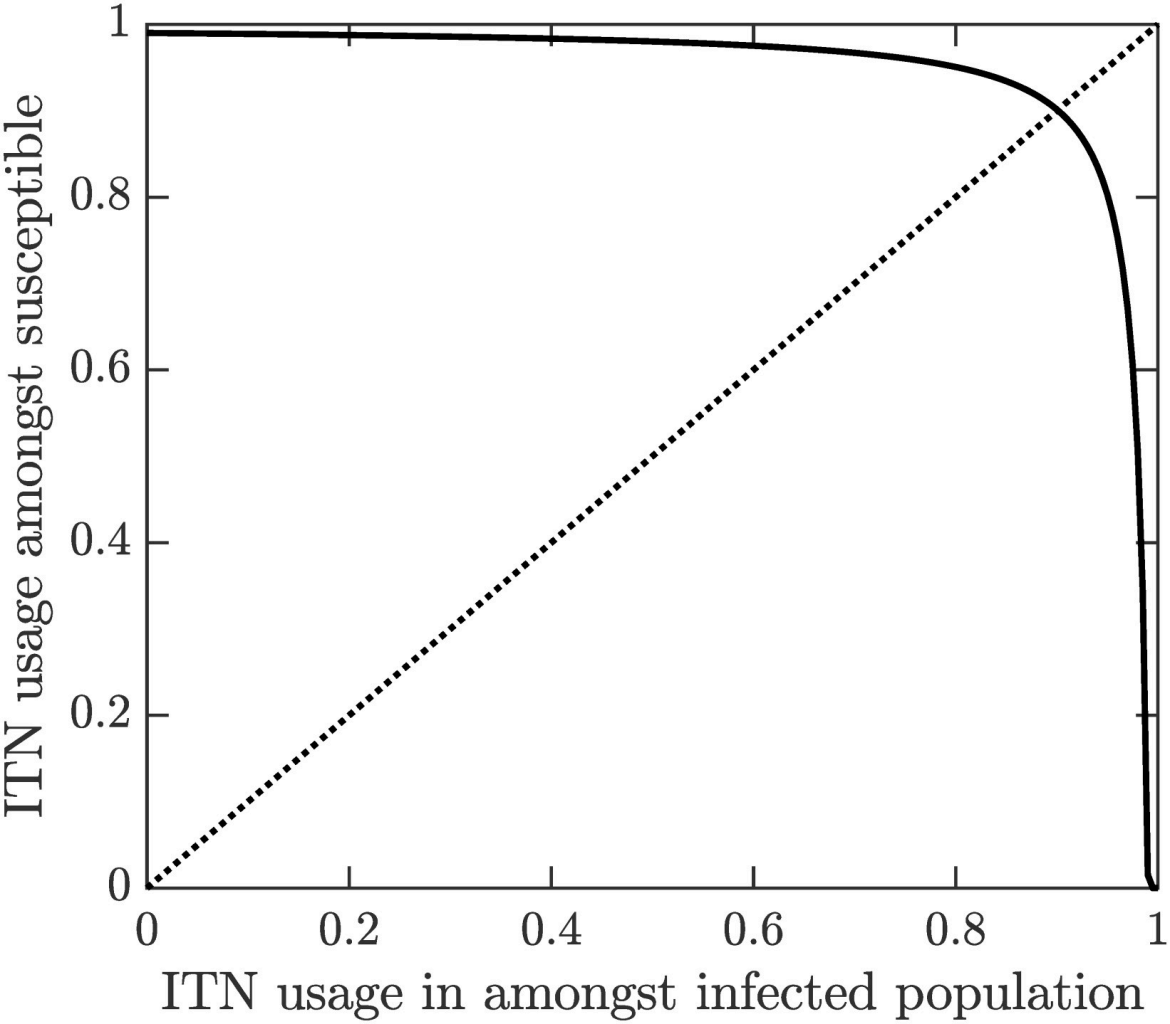
The explicit dependence of *p*_*HP*_, the level of ITN usage needed for VL elimination on the ITN usage amongst infected cases. We assume that *p*_*A*_ = *p*_*K*_ = *p*_*P*_ = *p*_*D*_ varies in [0, 1] while other parameters are as in Table 1. The dotted line represents *y* = *x*. The two curves intersect almost exactly at 0.9; i.e., if more than 90% of the population, including symptomatic and asymptomatic VL cases, used ITNs, VL could be eliminated.

We specifically investigated how *p*_*HP*_, the level of ITN usage needed for VL elimination, depends on ITN usages amongst the infected population. We set *p*_*A*_ = *p*_*K*_ = *p*_*P*_ = *p*_*D*_, varied these from 0 to 1 and calculated *p*_*HP*_ from (17). The results are shown in Fig 7. It follows that *p*_*HP*_ is staying quite high (> 90%) unless the ITN usage amongst the infected population itself is over 90%.

## Conclusions and discussion

In this paper we investigated a model of VL transmission. We built on the model developed in [17] and incorporated the human behavior component motivated by game theory [43].

To our knowledge, the presented model and [13] are the only two models of VL which also incorporate the game-theoretical component. As demonstrated in [55], that addition provides more insight and better predictions than standard compartmental epidemiological models. Compared to other models of VL such as [17, 20, 21] the current model allows us to predict not only what ITN coverage is needed to eliminate VL, but also what coverage can be realistically achieved.

The presented model was significantly simpler than [13] which built on a comprehensive, detailed and complex model of [21] informed by results of three different tests for VL. In contrast, our model builds on a much simpler model from [17]. Yet, the outcomes of both of the models were very similar, both qualitatively and quantitatively. Moreover, as shown in Table 3, both models also agree with data. More specifically, both models predict that to eliminate VL, 96%+ of the susceptible population should be using ITN. Both models are also in agreement that increasing the time between bites and reducing the number and/or the lifespan of sandflies are the most important control measures beyond the use of ITNs. These facts demonstrate that one does not need the most detailed model to make reasonably good predictions. This is in agreement with [22] who advocates for the use of simpler epidemiological models, especially for modeling diseases affecting developing countries in order to increase the tractability of the models.

Our model also predicts that if the whole population, including symptomatic, asymptomatic and dormant VL cases, use ITNs, then the reproduction number drops below 1 and, consequently, VL can be eliminated. This is, seemingly, in contradiction with the results of the KalaNet trial during which up to 91% of the population used ITNs without a significant reduction of VL cases [12]. However, as shown for example in [56], there is often a significant time-lag between the reduction of the reproduction number and the noticeable drop of disease cases. It is therefore entirely possible that if the 91% use of ITNs in the population was sustained, the VL case reduction would follow.

From the policy-making perspective, similar to [13], even our current simpler model suggests that instead of abandoning the use of ITNs completely, the ITN use should be combined with other intervention methods, including vector control.

There are several potential directions for future research. One can incorporate the fact that ITNs do not offer complete protection as the sandflies can bite the people outside of the ITNs. One can also incorporate the seasonality if the sandflies dynamics. Finally, the decisions to use ITNs could be modeled dynamically using the imitation dynamics as done in [57] and, for example, in [58–62].

## Supporting information

S1 File This file contains detailed calculations of mathematical formulas presented in the analysis section. (PDF)

S2 File This is the code we used to generate figures and perform the uncertainty and sensitivity analysis for the manuscript. (M)
