# Supplementary material for: A mathematical model of visceral leishmaniasis transmission and control: Impact of ITNs on VL prevention and elimination in the Indian subcontinent
Source: PLoS One. 2024 Oct 4;19(10):e0311314. doi: 10.1371/journal.pone.0311314 (PMC11452004; doi:10.1371/journal.pone.0311314)
Supplement: S1 File — (PDF) [file pone.0311314.s001.pdf]

Supporting information for
A mathematical model of visceral leishmaniasis transmission and control: impact of ITNs on VL prevention and elimination in the Indian subcontinent

Cameron Davis[1], Elizabeth R. Javor[2], Sonja I. Rebarber[3], Jan Rychtář[4*], Dewey Taylor[2],

1 Department of Mathematics, Fitchburg State University, Fitchburg, MA 01420, USA
2 Department of Mathematics, Rochester Institute of Technology, Rochester, NY 14623, USA
3 Department of Mathematics and Statistics, Swarthmore College, Swarthmore, PA 19081, USA
4 Department of Mathematics and Applied Mathematics, Virginia Commonwealth University, Richmond, VA 23284, USA

* rychtarj@vcu.edu

Detailed calculations

The model in Fig 1 yields the following system of ordinary differential equations.

$$\frac{dS}{dt} = \Lambda - (\lambda + \mu)S + \rho R \tag{1}$$
$$\frac{dE}{dt} = \lambda S - (\gamma_E + \mu)E \tag{2}$$
$$\frac{dI_A}{dt} = \gamma_E E - (\gamma_A + \mu)I_A \tag{3}$$
$$\frac{dI_K}{dt} = f_{AK}\gamma_A I_A - (\gamma_K + \mu + \mu_K)I_K \tag{4}$$
$$\frac{dI_D}{dt} = f_{AD}\gamma_A I_A + f_{KD}\gamma_K I_K - (\gamma_D + \mu)I_D \tag{5}$$
$$\frac{dI_P}{dt} = f_{AP}\gamma_A I_A + \gamma_D I_D - (\gamma_P + \mu)I_P \tag{6}$$
$$\frac{dR}{dt} = f_{AR}\gamma_A I_A + (1 - f_{KD})\gamma_K I_K + \gamma_P I_P - (\rho + \mu)R \tag{7}$$
$$\frac{dS_F}{dt} = \mu_F N_F - (\lambda_F + \mu_F)S_F \tag{8}$$
$$\frac{dI_F}{dt} = \lambda_F S_F - \mu_F I_F \tag{9}$$

where

$$\lambda = (1 - p)\beta i_F I_F, \tag{10}$$
$$\lambda_F = \beta\left(i_A(1 - p_A)I_A + i_K(1 - p_K)I_K + i_D(1 - p_D)I_D + i_P(1 - p_P)I_P\right). \tag{11}$$

Equilibria of the ODE system

The equilibria of the dynamics (1)-(9) can be obtained by solving the following system of algebraic equations:

$$
\begin{aligned}
0 &= \Lambda - (\lambda + \mu)S + \rho R && (12)\\
0 &= \lambda S - (\gamma_E + \mu)E && (13)\\
0 &= \gamma_E E - (\gamma_A + \mu)I_A && (14)\\
0 &= f_{AK}\gamma_A I_A - (\gamma_K + \mu + \mu_K)I_K && (15)\\
0 &= f_{AD}\gamma_A I_A + f_{KD}\gamma_K I_K - (\gamma_D + \mu)I_D && (16)\\
0 &= f_{AP}\gamma_A I_A + \gamma_D I_D - (\gamma_P + \mu)I_P && (17)\\
0 &= f_{AR}\gamma_A I_A + (1 - f_{KD})\gamma_K I_K + \gamma_P I_P - (\rho + \mu)R && (18)\\
0 &= \mu_F N_F - (\lambda_F + \mu_F)S_F && (19)\\
0 &= \lambda_F S_F - \mu_F I_F. && (20)
\end{aligned}
$$

The system (12)-(20) will be solved, similarly as in [1], by starting at compartment S and going downstream. By (13) and $\lambda = (1-p)\beta i_F I_F$,

$$
E^* = \frac{\lambda}{\mu + \gamma_E} S^* = (1-p)\beta i_F \frac{1}{\mu + \gamma_E} S^* I_F^*. \tag{21}
$$

Similarly, by (14)-(18), for every compartment $Comp \in (E, I_A, I_K, I_D, I_P, R)$ we have

$$
Comp^* = \lambda T_{Comp} S^* = (1-p)\beta i_F T_{Comp} S^* I_F^*. \tag{22}
$$

where the quantities T_{Comp} correspond to the expected time an individual spends in a compartment $Comp$ given it started in compartment E and are given by

$$
\begin{aligned}
T_E &= \frac{1}{\gamma_E + \mu}, && (23)\\
T_{I_A} &= \frac{\gamma_E T_E}{\gamma_A + \mu}, && (24)\\
T_{I_K} &= \frac{f_{AK}\gamma_A T_{I_A}}{\gamma_K + (\mu + \mu_K)}, && (25)\\
T_{I_D} &= \frac{f_{KD}\gamma_K T_{I_K} + f_{AD}\gamma_A T_{I_A}}{\gamma_D + \mu}, && (26)\\
T_{I_P} &= \frac{\gamma_D T_{I_D} + f_{AP}\gamma_A T_{I_A}}{\gamma_P + \mu}, && (27)\\
T_R &= \frac{(1 - f_{KD})\gamma_K T_{I_K} + \gamma_P T_{I_P} + f_{AR}\gamma_A T_{I_A}}{\rho + \mu}. && (28)
\end{aligned}
$$

Disease-free equilibrium

We assume that $E^0 = 0$. By (14), $I_A^0 = 0$. By (15), $I_K^0 = 0$. By (16), $I_D^0 = 0$. By (17), $I_P^0 = 0$. By (18), $R^0 = 0$. By (11), $\lambda_F = 0$. Thus by (19), $S_F^0 = N_F = n_F \frac{\Lambda}{\mu}$. Therefore by (20), $I_F^0 = 0$. Which means by (10), $\lambda = 0$. From (12), $S^0 = \frac{\Lambda}{\mu}$. So our disease free equilibrium is given by

$$
DFE^0 = \left(\frac{\Lambda}{\mu}, 0, 0, 0, 0, 0, 0, n_F \frac{\Lambda}{\mu}, 0 \right). \tag{29}
$$

Reproduction Number

The reproduction number, R_0, is found using the next-generation matrix method [2, 3]. The method proceeds as follows. We sort all VL carrying compartments as E, I_A, I_K, I_D, I_P, I_F. The vector $\mathscr{F}$ contains all secondary infections entering the compartments and $\mathscr{V}$ contains rates of all other transitions. Using $S_F = N_F = n_F N = n_F S$ and thus $S = \frac{S_F}{n_F}$, we get

$$\mathscr{F} = S_F \beta \begin{pmatrix} I_F \frac{i_F}{n_F}(1-p) \\ 0 \\ 0 \\ 0 \\ 0 \\ I_A i_A (1-p_A) + I_D i_D (1-p_D) + I_K i_K (1-p_K) + I_P i_P (1-p_P) \end{pmatrix}, \tag{30}$$

$$\mathscr{V} = \begin{pmatrix} -E(\gamma_E + \mu) \\ E\gamma_E - I_A(\gamma_A + \mu) \\ I_A f_{AK} \gamma_A - I_K(\gamma_K + \mu + \mu_K) \\ I_A f_{AD} \gamma_A + I_K f_{KD} \gamma_K - I_D(\gamma_D + \mu) \\ I_A f_{AP} \gamma_A + I_D \gamma_D - I_P(\gamma_P + \mu) \\ -I_F \mu_F \end{pmatrix}. \tag{31}$$

The Jacobian matrices are given by

$$F = S_F \beta \begin{pmatrix} 0 & 0 & 0 & 0 & 0 & \frac{i_F}{n_F}(1-p) \\ 0 & 0 & 0 & 0 & 0 & 0 \\ 0 & 0 & 0 & 0 & 0 & 0 \\ 0 & 0 & 0 & 0 & 0 & 0 \\ 0 & 0 & 0 & 0 & 0 & 0 \\ 0 & i_A(1-p_A) & i_K(1-p_K) & i_D(1-p_D) & i_P(1-p_P) & 0 \end{pmatrix}, \tag{32}$$

$$V = \begin{pmatrix} -(\gamma_E+\mu) & 0 & 0 & 0 & 0 & 0 \\ \gamma_E & -(\gamma_A+\mu) & 0 & 0 & 0 & 0 \\ 0 & f_{AK}\gamma_A & -(\gamma_K+\mu+\mu_K) & 0 & 0 & 0 \\ 0 & f_{AD}\gamma_A & f_{KD}\gamma_K & -(\gamma_D+\mu) & 0 & 0 \\ 0 & f_{AP}\gamma_A & 0 & \gamma_D & -(\gamma_P+\mu) & 0 \\ 0 & 0 & 0 & 0 & 0 & -\mu_F \end{pmatrix}. \tag{33}$$

Hence,

$$V^{-1} = \begin{pmatrix} -T_E & 0 & 0 & 0 & 0 & 0 \\ -T_{I_A} & -\frac{1}{\gamma_A+\mu} & 0 & 0 & 0 & 0 \\ -T_{I_K} & -\frac{f_{AK}\gamma_A}{(\gamma_A+\mu)(\gamma_K+(\mu+\mu_K))} & -\frac{1}{\gamma_K+\mu+\mu_K} & 0 & 0 & 0 \\ -T_{I_D} & v_{42} & -\frac{f_{KD}\gamma_K}{(\gamma_D+\mu)(\gamma_K+\mu+\mu_K)} & -\frac{1}{\gamma_D+\mu} & 0 & 0 \\ -T_{I_P} & v_{52} & v_{53} & -\frac{\gamma_D}{(\gamma_D+\mu)(\gamma_P+\mu)} & -\frac{1}{\gamma_P+\mu} & 0 \\ 0 & 0 & 0 & 0 & 0 & -\frac{1}{\mu_F} \end{pmatrix} \tag{34}$$

where

$$v_{42} = -\frac{\gamma_A \left(f_{AD}(\gamma_K+\mu+\mu_K)+f_{AD}\gamma_A f_{KD}\gamma_K\right)}{(\gamma_A+\mu)\,(\gamma_D+\mu)\,(\gamma_K+\mu+\mu_K)}, \tag{35}$$

$$v_{52} = -\frac{\gamma_A}{\gamma_A+\mu}\frac{(f_{AD}\gamma_D+f_{AP}\mu+f_{AP}\gamma_D)(\gamma_K+\mu+\mu_K)+f_{KD}f_{AK}\gamma_D\gamma_K}{(\gamma_D+\mu)\,(\gamma_K+\mu+\mu_K)\,(\gamma_P+\mu)}, \tag{36}$$

$$v_{53} = -\frac{f_{KD}\gamma_D\gamma_K}{(\gamma_D+\mu)\,(\gamma_K+\mu+\mu_K)\,(\gamma_P+\mu)}. \tag{37}$$

Thus,

$$FV^{-1} = -S_F\beta \begin{pmatrix} 0 & 0 & 0 & 0 & 0 & -\frac{i_F}{\mu_F n_F}(1-p) \\ 0 & 0 & 0 & 0 & 0 & 0 \\ 0 & 0 & 0 & 0 & 0 & 0 \\ 0 & 0 & 0 & 0 & 0 & 0 \\ 0 & 0 & 0 & 0 & 0 & 0 \\ -T_I & w_{62} & w_{63} & w_{64} & w_{65} & 0 \end{pmatrix} \tag{38}$$

where

$$T_I = i_A(1-p_A)T_{I_A} + i_K(1-p_K)T_{I_K} + i_D(1-p_D)T_{I_D} + i_P(1-p_P)T_{I_P} \tag{39}$$

and the values of w_{62}, w_{63}, w_{64} and w_{65} are irrelevant. The spectral radius of FV^{-1} is

$$\rho(FV^{-1}) = S_F\beta\sqrt{(1-p)\frac{i_F}{\mu_F n_F}T_I}. \tag{40}$$

There are humans and flies involved in the transmission cycle. Hence, a single infection in otherwise susceptible population will cause

$$R_0(\overline{p}) = \rho(FV^{-1})^2 = (1-p)\beta^2 n_F \left(\frac{\Lambda}{\mu}\right)^2 i_F \frac{1}{\mu_F} T_I \tag{41}$$

secondary infections [3].

Endemic equilibrium

By looking at all human compartments and balancing what is going in and out (i.e. adding all equations (12)–(20)), we get

$$\Lambda = \mu S + \mu E + \mu I_A + (\mu+\mu_K)I_K + \mu I_D + \mu I_P + \mu R. \tag{42}$$

By (22)-(28) and (10),

$$\Lambda = \mu S + \mu T_E\lambda S + \mu T_{I_A}\lambda S + (\mu+\mu_K)T_{I_K}\lambda S + \mu T_{I_D}\lambda S + \mu T_{I_P}\lambda S + \mu T_R\lambda S \tag{43}$$

$$= \mu S(1+\lambda(T_E + T_{I_A} + \frac{(\mu+\mu_K)}{\mu}T_{I_K} + T_{I_D} + T_{I_P} + T_R)) \tag{44}$$

$$= \mu S(1+\lambda T_{cycle}) \tag{45}$$

$$= \mu S(1+(1-p)\beta i_F T_{Cycle} I_F^*), \tag{46}$$

where

$$T_{Cycle} = T_E + T_{I_A} + \frac{(\mu + \mu_K)}{\mu} T_{I_K} + T_{I_D} + T_{I_P} + T_R. \tag{47}$$

Solving for S^* gives

$$S^* = \frac{\frac{\Lambda}{\mu}}{1 + (1-p)\beta i_F T_{Cycle} I_F^*}. \tag{48}$$

By (11), (22), and (24)-(27),

$$\begin{aligned}
\lambda_F &= \beta\left(i_A(1-p_A)I_A^* + i_K(1-p_K)I_K^* + i_D(1-p_D)I_D^* + i_P(1-p_P)I_P^*\right) && (49)\\
&= \beta\left(i_A(1-p_A)\lambda T_A S^* + i_K(1-p_K)\lambda T_K S^* + i_D(1-p_D)\lambda T_D S^* + i_P(1-p_P)\lambda T_P S^*\right) && (50)\\
&= \beta\lambda S^*\left(i_A(1-p_A)T_{I_A} + i_K(1-p_K)T_{I_K} + i_D(1-p_D)T_{I_D} + i_P(1-p_P)T_{I_P}\right) && (51)\\
&= \beta\lambda S^* T_I. && (52)
\end{aligned}$$

Hence, by (48), (10), and (41)

$$\begin{aligned}
\lambda_F = \beta\lambda S^* T_I &= \beta(1-p)\beta i_F I_F^* \frac{\frac{\Lambda}{\mu}}{1+(1-p)\beta i_F I_F^* T_{Cycle}} T_I && (53)\\
&= \frac{\beta(\frac{\Lambda}{\mu})T_I I_F^* \mu_F R_0}{\beta n_F(\frac{\Lambda}{\mu})(\frac{\Lambda}{\mu})T_I + I_F \mu_F T_{Cycle} R_0}. && (54)
\end{aligned}$$

By (20), $S_F^* = \frac{\mu_F}{\lambda_F} I_F^*$. and thus, by (54),

$$\begin{aligned}
N_F^* = S_F^* + I_F^* &= \frac{\mu_F}{\lambda_F} I_F^* + I_F^* && (55)\\
&= \frac{\beta n_F(\frac{\Lambda}{\mu})(\frac{\Lambda}{\mu})T_I + I_F^* \mu_F T_{Cycle} R_0}{\beta(\frac{\Lambda}{\mu})T_I R_0} + I_F^* && (56)
\end{aligned}$$

Solving for I_F^* yields

$$I_F^* = \frac{n_F \beta \Lambda^2 T_I}{\mu^2 T_{Cycle} \mu_F + \mu\beta\Lambda T_I}\left(1 - \frac{1}{R_0}\right). \tag{57}$$

Once I_F^* is calculated from (57), we can calculate S_F^* and S^* as

$$\begin{aligned}
S^* &= \frac{(\frac{\Lambda}{\mu})}{(1+(1-p)\beta i_F T_{Cycle} I_F^*)}, && (58)\\
\lambda_F^* &= \beta\lambda S^* T_I, && (59)\\
S_F^* &= \frac{\mu_F}{\lambda_F} I_F^*. && (60)
\end{aligned}$$

Finally, using above, we can also find all the human compartments as below

$$E^* = (1-p)\beta i_F T_E S^* I_F^*, \tag{61}$$
$$I_A^* = (1-p)\beta i_F T_{I_A} S^* I_F^*, \tag{62}$$
$$I_K^* = (1-p)\beta i_F T_{I_K} S^* I_F^*, \tag{63}$$
$$I_D^* = (1-p)\beta i_F T_{I_D} S^* I_F^*, \tag{64}$$
$$I_P^* = (1-p)\beta i_F T_{I_P} S^* I_F^*, \tag{65}$$
$$R^* = (1-p)\beta i_F T_R S^* I_F^*. \tag{66}$$

References

1. Fortunato AK, Glasser CP, Watson JA, Lu Y, Rychtář J, Taylor D. Mathematical modelling of the use of insecticide-treated nets for elimination of visceral leishmaniasis in Bihar, India. Royal Society Open Science. 2021;8(6):201960.

2. Diekmann O, Heesterbeek JAP, Metz JA. On the definition and the computation of the basic reproduction ratio R 0 in models for infectious diseases in heterogeneous populations. Journal of Mathematical Biology. 1990;28(4):365–382.

3. van den Driessche P, Watmough J. Reproduction numbers and sub-threshold endemic equilibria for compartmental models of disease transmission. Mathematical Biosciences. 2002;180:29–48. doi:10.1016/S0025-5564(02)00108-6.
